# New Marine Sterols from a Gorgonian *Pinnigorgia* sp.

**DOI:** 10.3390/molecules22030393

**Published:** 2017-03-03

**Authors:** Yu-Chia Chang, Tsong-Long Hwang, Chih-Hua Chao, Ping-Jyun Sung

**Affiliations:** 1 National Museum of Marine Biology & Aquarium, Pingtung 944, Taiwan; jay0404@gmail.com; 2 Doctoral Degree Program in Marine Biotechnology, National Sun Yat-sen University & Academia Sinica, Kaohsiung 804, Taiwan; 3 Greenhouse Systems Technology Center, Central Region Campus, Industrial Technology Research Institute, Nantou 540, Taiwan; 4 Graduate Institute of Natural Products, College of Medicine and Chinese Herbal Medicine Research Team, Healthy Aging Research Center, Chang Gung University, Taoyuan 333, Taiwan; htl@mail.cgu.edu.tw; 5 Research Center for Chinese Herbal Medicine, Research Center for Food and Cosmetic Safety, and Graduate Institute of Health Industry Technology, College of Human Ecology, Chang Gung University of Science and Technology, Taoyuan 333, Taiwan; 6 Department of Anesthesiology, Chang Gung Memorial Hospital, Taoyuan 333, Taiwan; 7 School of Pharmacy, China Medical University, Taichung 404, Taiwan; chchao@mail.cmu.edu.tw; 8 Chinese Medicine Research and Development Center, China Medical University Hospital, Taichung 404, Taiwan; 9 Graduate Institute of Marine Biology, National Dong Hwa University, Pingtung 944, Taiwan; 10 Graduate Institute of Natural Products, Kaohsiung Medical University, Kaohsiung 807, Taiwan; 11 Department of Marine Biotechnology and Resources, National Sun Yat-sen University, Kaohsiung 804, Taiwan

**Keywords:** gorgonian, *Pinnigorgia*, anti-inflammatory, superoxide anion, elastase, pubinernoid A, loliolide

## Abstract

Continuous chemical investigation of the gorgonian coral *Pinnigorgia* sp. resulted in the isolation of two new sterols, 5α,6α-epoxy-(22*E*,24*R*)-3β,11-dihydroxy-9,11-secoergosta-7-en-9-one (**1**) and (22*R*)-acetoxy-(24*ξ*)-ergosta-5-en-3β,25-diol (**2**). The structures of sterols **1** and **2** were elucidated using spectroscopic methods. Sterol **1** displayed inhibitory effects on the generation of superoxide anions and the release of elastase by human neutrophils with IC_50_ values of 8.65 and 5.86 μM, respectively. The structure of a known metabolite, pubinernoid A (**3**), is revised as (+)-loliolide (**4**).

## 1. Introduction

**Figure 1:**
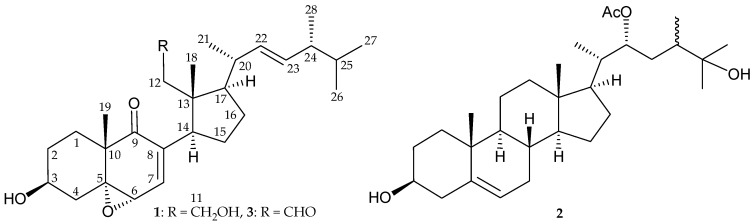
Chemical structures of 5α,6α-epoxy-(22*E*,24*R*)-3β,11-dihydroxy-9,11-secoergosta-7-en-9- one (**1**), (22*R*)-acetoxy-(24*ξ*)-ergosta-5-en-3β,25-diol (**2**), and 3-*O*-deacetylluffasterol B (**3**) [7].

Gorgonian corals belonging to the genus *Pinnigorgia* have proven to be a rich source of sterols with unusual structural features [1,2,3,4,5,6]. In continuation of our effort to discover new natural products of this organism, its ethyl acetate extract exhibited anti-inflammatory activities by inhibiting the expression of superoxide anions and elastase by human neutrophils with IC_50_ values of 1.89 and 1.57 μg/mL, respectively. Two new sterols, 5α,6α-epoxy-(22*E*,24*R*)-3β,11-dihydroxy-9,11-secoergosta-7-en-9-one (**1**) and (22*R*)-acetoxy-(24*ξ*)-ergosta-5-en-3β,25-diol (**2**) (Figure 1 and Supplementary Figures S1–S14), were isolated. The structures of these two sterols were established by spectroscopic analyses and sterol **1** was found to display anti-inflammatory activity.

## 2. Results and Discussion

The new sterol, 5α,6α-epoxy-(22*E*,24*R*)-3β,11-dihydroxy-9,11-secoergosta-7-en-9-one (**1**), was isolated as colorless oil. The high-resolution electrospray ionization mass spectrum (HRESIMS) showed a signal at *m/z* 467.31308 (calcd. for C_28_H_44_O_4_ + Na, 467.31373), and therefore the molecular formula of **1** was determined to be C_28_H_44_O_4_ (7° of unsaturation degrees). The ^13^C and distortionless enhancement polarization transfer (DEPT) spectrum of **1** showed that this compound has 28 carbons including six methyls, seven sp^3^ methylenes, seven sp^3^ methines, an sp^3^ oxygenated tertiary carbon, two sp^3^ quaternary carbons, three sp^2^ methines, an sp^2^ tertiary carbon and a ketonic carbonyl (Table 1). The IR spectrum of **1** revealed the presence of hydroxy (ν_max_ 3398 cm^−1^) and α,β-unsaturated ketonic carbonyl (ν_max_ 1682 cm^−1^) groups. The latter structural feature was confirmed by the presence of signals at δ_C_ 201.9 (C-9), 141.4 (C-8) and 139.3 (CH-7) in the ^13^C-NMR spectrum. A disubstituted olefin was identified from the signals of carbons at δ_C_ 134.4 (CH-22) and 133.0 (CH-23) and was confirmed by two olefin proton signals at δ_H_ 5.21 (1H, dd, *J* = 15.2, 6.4 Hz, H-23) and 5.24 (1H, dd, *J* = 15.2, 6.8 Hz, H-22) (Table 1). Four doublets at δ_H_ 1.03 (3H, *J* = 6.8 Hz), 0.91 (3H, *J* = 6.8 Hz), 0.83 (3H, *J* = 7.2 Hz) and 0.82 (3H, *J* = 6.8 Hz) were due to the Me-21, Me-28, Me-26 and Me-27 groups, respectively. Two sharp singlets for H_3_-18 and H_3_-19 appeared at δ_H_ 0.68 and 1.25, respectively. A trisubstituted epoxide was elucidated from the signals of an oxygenated tertiary carbon at δ_C_ 63.2 (C-5) and an oxymethine at δ_C_ 53.5 (CH-6); and further confirmed by the proton signal of a methine doublet at δ_H_ 3.39 (1H, d, *J* = 4.8 Hz, H-6). On the basis of the unsaturation data overall, **1** was concluded to be a secosterol molecule possessing four rings.

**Figure 2:**
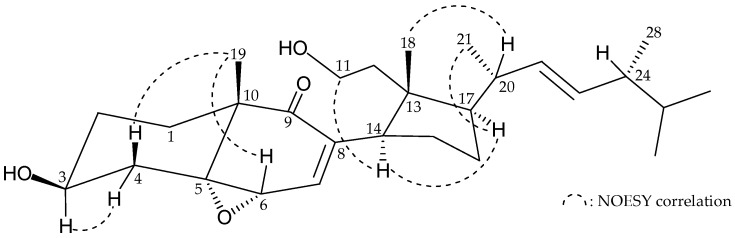
Selected NOESY correlations observed for **1**.

From the ^1^H-NMR coupling information and ^1^H–^1^H correlation spectroscopy (COSY) of **1** (Table 1), the following correlations were revealed: H_2_-1/H_2_-2/H-3/H_2_-4, H-6/H-7, H_2_-11/H_2_-12, H-14/H_2_-15/H_2_-16/H-17/H-20/H-22/H-23/H-24/H-25/H_3_-26, H-20/H_3_-21, H-24/H_3_-28 and H-25/H_3_-27. These data, together with the key heteronuclear multiple bond coherence (HMBC) correlations between H_2_-1, H_2_-4, H_3_-19/C-5; H-6/C-8; H-7, H_3_-19/C-9; H_2_-2, H_3_-19/C-10; and H_2_-12, H_2_-15, H_3_-18/ C-13 (Table 1), all the information allowed determination of the carbon skeleton of **1**. The stereochemistry of **1** was elucidated by analysis of the results of a nuclear Overhauser effect spectroscopy (NOESY) experiment. Assuming the β-orientation of H_3_-18 and H_3_-19, H-14 was found to exhibit correlations with H-11a (δ_H_ 3.81) and H-17, but not with H_3_-18, indicating that this proton was of an α-orientation at C-14. In addition, the main NOESY correlation for **1** were interactions between H-3/H-4α, H-4β/H_3_-19, H-6/H_3_-19, H-17/H_3_-21 and H_3_-18/H-20; thus, the 3-hydroxy and 5,6-epoxy groups in **1** should be positioned on the β- and α-face, respectively (Figure 2).

**Figure 3:**
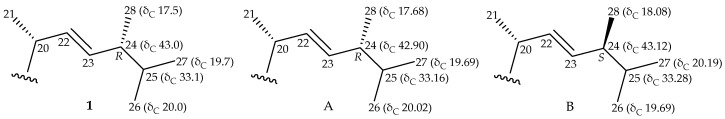
The ^13^C-NMR chemical shifts of the side-chain of secosterol **1**, (22*E*,24*R*)-24-methyl-cholesta-5,22-dien-3β-ol (A) and (22*E*,24*S*)-24-methylcholesta-5,22-dien-3β-ol (B) [8].

A large coupling constant observed between H-22 and H-23 (*J* = 15.2 Hz) supported a *trans* relationship between H-22 and H-23. The configuration of C-24 was suggested to be *R* on the basis of the ^13^C-NMR chemical shift of C-28 (δ_C_ 17.5). It was reported that the ^13^C-NMR value of C-28 resonates at δ_C_ 17.68 ppm in the 24*R* epimer of a known sterol, (22*E*,24*R*)-24-methylcholesta-5,22-dien-3β-ol, with the same chain, and the 24*S* epimer, (22*E*,24*S*)-24-methylcholesta-5,22-dien-3β-ol, has a relative 0.4 ppm downfield chemical shift (Figure 3) [8].

It was found that the NMR data of 1 were similar to those of a known 9,11-secosterol derivative, 3-*O*-deacetylluffasterol B (3) (Figure 1), isolated from the sponge *Spongia agaricina* [7], except that the signals corresponding to the 11-hydroxy group in 1 were replaced by signals for an aldehyde group in 3 [7] (Table 1). Furthermore, by comparison of the NMR data of 1 with those of 3, we found that the ^13^C NMR chemical shifts of methines C-20 and C-24 for 3 (δ_C_ 43.0 and 38.8, respectively) should be interchangeable by comparison with those of 1 (δ_C_ 38.8 and 43.0, respectively) (Table 1), which was further confirmed by 2D NMR experiments. In a previous study, the structure of 1 as presented in this paper had been reported [9]. However, by comparison of the NMR data of 1 with those of reported data, we found that the NMR data (^1^H and ^13^C) for this compound differ significantly from those of 1 that reported herein (Table 1), because the structure of 1 has been established by extensive spectroscopic analysis, particularly with 2D NMR experiments. The authors suggested that the compound which was reported to possess the same structure as that of 1 in Reference [9] should be re-examined.

(22*R*)-Acetoxy-(24*ξ*)-ergosta-5-en-3β,25-diol (**2**) was isolated as a colorless needles and its molecular formula was established as C_30_H_50_O_4_ (6° of unsaturation) by HRESIMS at *m/z* 497.36015 (calcd. for C_30_H_50_O_4_ + Na, 497.36193). The IR spectrum of **2** indicated the presence of hydroxy (ν_max_ 3414 cm^−1^) and ester carbonyl (ν_max_ 1730 cm^−1^) groups. The whole series of spectroscopic data obtained from 1D and 2D NMR experiments (Table 2) clearly indicated that sterol **2** had the same core rings A−D and side chain as those of known sterols, 22(*R*),28-oxido-24*ξ*-methylcholest-5-en-3β,25,28-triol (lobophytosterol) and (22*R*)-5β,6β-epoxy-24*ξ*-methylcholestan-3β,22(*R*),25-triol diacetate, respectively [10]. The ^1^H-^1^H COSY and HMBC correlations observed fully supported the locations of the functional groups, and, hence, (22*R*)-acetoxy-(24*ξ*)-ergosta-5-ene-3β,25-diol (**2**) was assigned as structure **2**, with the same relative configurations as 22(*R*),28-oxido-24*ξ*-methylcholest-5-en-3β,25,28-triol in the core rings A–D. The configuration of the C-22 stereogenic center was assigned as *R* on the basis of the NMR chemical shifts of C-22 oxymethine (δ_H_ 5.02, 1H, ddd, *J* = 10.8, 2.8, 2.4 Hz, H-22; δ_C_ 78.3, CH-22). It was reported that the ^1^H- and ^13^C=NMR values of C-22 oxymethine (δ_H_ 4.99, 1H, dt, *J* = 10.2, 2.5 Hz, H-22; δ_C_ 78.3, CH-22) in a 22*R* epimer of a known sterol, 5β,6β-epoxy-24*ξ*-methylcholestan-3β,22(*R*),25-triol diacetate [10], was found to possess the same side chain as that of **2**. The proton coupling constants and NMR chemical shift data also further supported these findings, though the configuration of C-24 was not determined at this stage.

The sterol analogues isolated from *Pinnigorgia* sp. were found to display interesting anti-inflammatory activities [1,2,3,5,6]. Based on these findings, the anti-inflammatory testing of sterols **1** and **2** were assayed and **1** showed inhibitory effects on the generation of superoxide anions and the release of elastase, respectively, by human neutrophils (Table 3).

**Figure 4:**
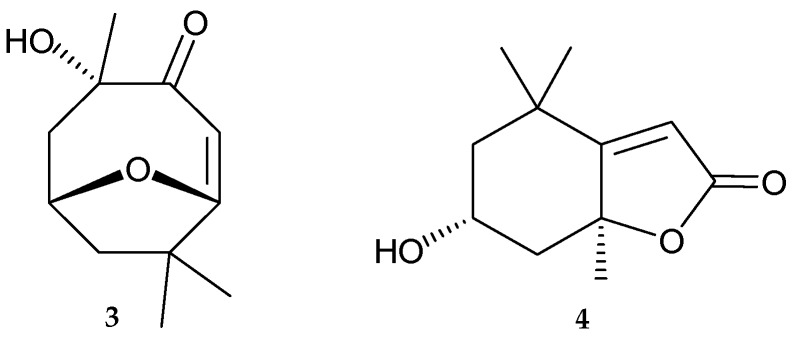
Chemical structures of pubinernoid A (**3**) and (+)-loliolide (**4**).

In a previous study, we reported the isolation and structure elucidation of a natural product, pubinernoid A (**3**), from *Pinnigorgia* sp. [11] and this compound which has been previously isolated from a traditional Chinese medicinal plant *Schisandra pubescens* var. *pubinervis* [12]. Based on the detailed spectroscopic analysis and by comparing the ^1^H- and ^13^C-NMR chemical shifts in **3** with those of known carotenoid metabolites, (±)-loliolide, [13,14,15,16,17,18,19], the structure of pubinernoid A (**3**) should be revised as (+)-loliolide as presented in **4** (Figure 4). Because (±)-loliolide were synthesized by chemical methods [18] and the structure of (–)-loliolide was established by X-ray diffraction analysis [19], the structure of pubinernoid A (**3**) should be revised as (+)-loliolide (**4**).

## 3. Experimental Section

### 3.1. General Experimental Procedures

Optical rotations were measured on a Jasco P-1010 digital polarimeter (Japan Spectroscopic Corporation, Tokyo, Japan). Infrared spectra were recorded on a Jasco FT/IR-4100 spectrometer (Japan Spectroscopic Corporation); peaks are reported in cm^–1^. The NMR spectra were recorded on a 400 MHz Varian Mercury Plus NMR spectrometer (Varian Inc., Palo Alto, CA, USA), using the residual CHCl_3_ signal (δ_H_ 7.26 ppm) as an internal standard for ^1^H-NMR and CDCl_3_ (δ_C_ 77.1 ppm) for ^13^C-NMR; coupling constants (J) are given in Hz. ESIMS and HRESIMS were recorded using a Bruker 7 Tesla solariX FTMS system (Bruker, Bremen, Germany). Column chromatography was performed on silica gel (230–400 mesh, Merck, Darmstadt, Germany). TLC was carried out on precoated Kieselgel 60 F_254_ (0.25 mm, Merck); spots were visualized by spraying with 10% H_2_SO_4_ solution followed by heating. Normal-phase HPLC (NP-HPLC) was performed using a system comprised of a Hitachi L-7110 pump (Hitachi Ltd., Tokyo, Japan) and a Rheodyne 7725 injection port (Rheodyne LLC, Rohnert Park, CA, USA). A semi-preparative normal-phase column (Supelco Ascentis Si Cat #:581515-U, 25 cm × 21.2 mm, 5 μm, Sigma-Aldrich, St. Louis, MO, USA) was used for NP-HPLC. Reversed-phase HPLC (RP-HPLC) was performed using a system comprised of a Hitachi L-2130 pump (Hitachi Ltd., Tokyo, Japan), a Hitachi L-2455 photodiode array detector (Hitachi Ltd., Tokyo, Japan) and a Rheodyne 7725 injection port (Rheodyne LLC., Rohnert Park, CA, USA). A reverse phase column (Luna 5 μm C18(2) 100 Å, AXIA Packed, 25 cm × 21.2 mm, Phenomenex Inc., Torrance, CA, USA) was used for RP-HPLC.

### 3.2. Animal Material

Specimens of the gorgonian corals *Pinnigorgia* sp. were collected by hand using scuba off the coast of Green Island, Taiwan in August 2012 and stored in a freezer until extraction. A voucher specimen (NMMBA-TW-GC-2012-130) was deposited in the National Museum of Marine Biology & Aquarium, Taiwan. This organism was identified by comparison with previous descriptions [20]. 

### 3.3. Extraction and Separation

Sliced bodies of *Pinnigorgia* sp. (wet weight 1.98 kg; dry weight 0.86 kg) were extracted with ethyl acetate (EtOAc) at room temperature. The EtOAc extract (84.9 g) was partitioned between methanol (MeOH) and *n*-hexane. The MeOH layer (12.6 g) was separated on Sephadex LH-20 and eluted using a mixture of dichloromethane (DCM) and MeOH (1:1) to yield 7 subfractions A–G. Fraction F was separated by silica gel column chromatography and eluted using *n*-hexa ne/acetone (stepwise, 1:1–pure acetone) to afford 8 subfractions F1–F8. Fraction F2 was purified by silica gel column chromatography and eluted using *n*-hexane/acetone (stepwise, 9:1–pure acetone) to yield 13 subfractions F2A–F2M. Fraction F2H was purified by NP-HPLC using a mixture of *n*-hexane/EtOAc (1:1) to afford 14 subfractions F2H1–F2F14. Fraction F2H12 was re-purified by RP-HPLC using a mixture of MeOH/H_2_O (90:10, 4.0 mL/min flow rate) to yield **1** (2.8 mg). Fraction F2D was purified by NP-HPLC using a mixture of *n*-hexane/EtOAc (3:1) to afford 17 subfractions F2D1–F2D117. Fraction F2D12 was re-purified by RP-HPLC using MeOH (1.5 mL/min flow rate) to yield **2** (2.6 mg).

*5α,6α-Epoxy-(22E,24R)-3β,11-dihydroxy-9,11-secoergosta-7-en-9-one* (**1**): colorless oil; [α]${}_{D}^{25}$ −35 (*c* 0.9, CHCl_3_); IR (neat) ν_max_ 3398, 1682 cm^−1^; ^1^H (400 MHz, CDCl_3_) and ^13^C (100 MHz, CDCl_3_) NMR data (see Table 1); ESIMS *m*/*z* 467 [M + Na]^+^; HRESIMS *m*/*z* 467.31308 (calcd. for C_28_H_44_O_4_ + Na, 467.31373).

*(22R)-Acetoxy-(24ξ)-ergosta-5-en-3β,25-diol* (**2**): colorless needles; mp. 130−132 °C; [α]${}_{D}^{27}$ −111 (*c* 0.7, CHCl_3_); IR (neat) ν_max_ 3414, 1730 cm^−1^; ^1^H (400 MHz, CDCl_3_) and ^13^C (100 MHz, CDCl_3_) NMR data (see Table 2); ESIMS *m*/*z* 497 [M + Na]^+^; HRESIMS *m*/*z* 497.36015 (calcd. for C_30_H_50_O_4_ + Na, 497.36193).

### 3.4. Generation of Superoxide Anions and Release of Elastase by Human Neutrophils 

Human neutrophils were obtained by means of dextran sedimentation and Ficoll centrifugation. Measurements of superoxide anion generation and elastase release were carried out according to previously described procedures [21,22]. Briefly, superoxide anion production was assayed by monitoring the superoxide dismutase-inhabitable reduction of ferricytochrome *c*. Elastase release experiments were performed using MeO-Suc-Ala-Ala-Pro-Valp-nitroanilide as the elastase substrate.

## 4. Conclusions

Our further studies on *Pinnigorgia* sp. for the extraction of natural substances have led to the isolation of two new marine sterols, 5α,6α-epoxy-(22*E*,24*R*)-3β,11-dihydroxy-9,11-secoergosta-7-en-9-one (**1**) and (22*R*)-acetoxy-(24*ξ*)-ergosta-5-en-3β,25-diol (**2**), and **1** showed potentially anti- inflammatory activity. These results suggested that continuing investigation of new secondary metabolites together with the potentially useful bioactive substances from *Pinnigorgia* sp. are worthwhile for future drug development.

## Figures and Tables

**Table 1:** ^1^H- and ^13^C-NMR data, ^1^H-^1^H COSY, and HMBC correlations for secosterol **1** and the ^1^H- and ^13^C-NMR data for 3-*O*-deacetylluffasterol B (**3**).

Position	1				3	
	δ_H_ (*J* in Hz) ^a^	δ_C_^b^	^1^H-^1^H	HMBC	δ_H_ (*J* in Hz)^c^	δ_C_^c^
1a/b	2.09 m; 1.72 m	27.8, CH_2_	H_2_-2	C-5		27.8, CH_2_
2a/b	2.09 m; 1.65 m	30.5, CH_2_	H_2_-1, H-3	C-10	2.09 m; 1.68 m	30.5, CH_2_
3	3.98 m	68.3, CH	H_2_-2, H_2_-4	n. o. ^d^	3.98 m	68.3, CH
4α	1.57 m	37.4, CH_2_	H-3, H-4β	C-2, -3, -5	1.56 m	37.5, CH_2_
β	2.18 dd (12.8, 11.6)		H-3, H-4α	C-3	2.18 m	
5		63.2, C				63.5, C
6	3.39 d (4.8)	53.5, CH	H-7	C-7, -8	3.40 d (4.6)	53.5, CH
7	6.81 d (4.8)	139.3, CH	H-6	C-5, -6, -9, -14	6.84 dd (4.6, 1.0)	139.7, CH
8		141.4, C				140.5, C
9		201.9, C				200.6, C
10		45.6, C				45.4, C
11a	3.81 ddd (10.4, 10.4, 6.0)	59.1, CH_2_	H-11b, H_2_-12	n. o.	9.88 dd (3.8, 1.7)	203.4, CH
b	3.68 ddd (10.4, 8.8, 6.0)		H-11a, H_2_-12	n. o.		
12a	1.61 m	40.4, CH_2_	H_2_-11, H-12b	n. o.	2.27 dd (15.9, 3.8)	50.8, CH_2_
b	1.12 m		H_2_-11, H-12a	C-11, -13, -17	2.00 dd (15.9, 1.7)	
13		46.1, C				46.3, C
14	3.37 dd (10.8, 8.0)	43.8, CH	H_2_-15	n. o.	3.51 dd (10.3, 9.2)	45.0, CH
15a/b	1.69–1.56 m	26.9, CH_2_	H-14, H_2_-16	C-13, -14	1.78 m; 1.71 m	26.7, CH_2_
16a/b	1.69 m; 1.44 m	25.4, CH_2_	H_2_-15, H-17	n. o.		25.8, CH_2_
17	1.74 m	49.6, CH	H_2_-16, H-20	n. o.		51.9, CH
18	0.68 s	17.8, CH_3_		C-12, -13, -14, -17	0.76 s	17.1, CH_3_
19	1.25 s	21.4, CH_3_		C-1, -5, -9, -10	1.21 s	20.0, CH_3_
20	2.15 m	38.8, CH	H-17, H_3_-21, H-22	n. o.	2.18 m	43.0, CH
21	1.03 d (6.8)	21.4, CH_3_	H-20	C-17, -20, -22	1.00 d (6.8)	19.7, CH_3_
22	5.24 dd (15.2, 6.8)	134.4, CH	H-20, H-23	C-20, -24	5.20 dd (17.6, 7.4)	133.4, CH
23	5.21 dd (15.2, 6.4)	133.0, CH	H-22, H-24	C-20, -24	5.24 dd (17.6, 7.4)	134.0, CH
24	1.86 m	43.0, CH	H-23, H-25, H_3_-28	C-22, -23, -25	1.87 m	38.8, CH
25	1.47 m	33.1, CH	H-24, H_3_-26, H_3_-27	C-23, -24, -28	1.47 m	33.2, CH
26	0.83 d (7.2)	20.0, CH_3_	H-25	C-24, -25, -27	0.82 d (6.8)	21.9, CH_3_
27	0.82 d (6.8)	19.7, CH_3_	H-25	C-24, -25, -26	0.83 d (6.8)	21.1, CH_3_
28	0.91 d (6.8)	17.5, CH_3_	H-24	C-23, -24, -25	0.91 d (7.0)	17.8, CH_3_

^a^ Spectra recorded at 400 MHz in CDCl_3_. ^b^ Spectra recorded at 100 MHz in CDCl_3_. ^c^ Selected ^1^H-NMR and ^13^C-NMR data were reported by Rueda et al. (see ref. [7]). These data were recorded at 400 MHz for ^1^H and 100 MHz for ^13^C in CDCl_3_. ^d^ n. o. = not observed.

**Table 2:** ^1^H (400 MHz, CDCl_3_) and ^13^C (100 MHz, CDCl_3_) NMR data and ^1^H-^1^H COSY and HMBC correlations for sterol **2**.

Position	δ_H_ (*J* in Hz)	δ_C_, Multiple	^1^H-^1^H COSY	HMBC
1a/b	1.84 m; 1.06 m	37.2, CH_2_	H_2_-2	C-2, -5
2a/b	1.84 m; 1.51 m	31.6, CH_2_	H_2_-1, H-3	n. o. ^a^
3	3.52 m	71.8, CH	H_2_-2, H_2_-4	n. o.
4a/b	2.30 m; 2.24 m	42.3, CH_2_	H-3	C-2, -3, -5, -6, -10
5		140.7, C		
6	5.35 d (5.2)	121.6, CH	H_2_-7	C-4, -7, -8, -10
7a/b	1.97 m; 1.53 m	31.8, CH_2_	H-6, H-8	C-6, -14
8	1.46 m	31.9, CH	H_2_-7, H-9, H-14	C-14
9	0.92 m	50.1, CH	H-8, H_2_-11	C-7, -8
10		36.5, C		
11a/b	1.50–1.40 m	21.1, CH_2_	H-9, H_2_-12	C-9
12a/b	1.97 m; 1.17 m	39.7, CH_2_	H_2_-11	n. o.
13		42.7, C		
14	0.98 m	56.3, CH	H-8, H_2_-15	n. o.
15a/b	1.56 m; 1.07 m	24.3, CH_2_	H-14, H_2_-16	C-14
16a/b	1.81 m; 1.53 m	27.2, CH_2_	H_2_-15, H-17	n. o.
17	1.15 m	53.1, CH	H_2_-16, H-20	C-12, -20
18	0.67 s	11.9, CH_3_		C-12, -13, -14, -17
19	1.00 s	19.4, CH_3_		C-1, -5, -9
20	1.75 m	39.8, CH	H-17, H_3_-21, H-22	n. o.
21	0.93 d (7.2)	13.0, CH_3_	H-20	C-17, -20, -22
22	5.02 ddd (10.8, 2.8, 2.4)	78.3, CH	H-20, H_2_-23	n. o.
23a/b	1.84 m; 1.15 m	29.3, CH_2_	H-22, H-24	C-20, -22
24	1.43 m	43.1, CH	H_2_-23, H_3_-28	n. o.
25		73.6, C		
26	1.13 s	25.0, CH_3_		C-24, -25, -27
27	1.20 s	28.4, CH_3_		C-24, -25, -26
28	0.91 d (7.2)	16.9, CH_3_	H-24	C-23, -24, -25
22-OAc		171.0, C		
	2.04, s	21.6, CH_3_		Acetate carbonyl

^a^ n. o. = not observed.

**Table 3:** Inhibitory effects of sterols **1** and **2** on superoxide anion generation and elastase release by human neutrophils in response to fMet-Leu-Phe/Cytochalastin B.

Compound	Superoxide Anions	Elastase Release
	IC_50_ (μM) ^a^	IC_50_ (μM)
**1**	8.65 ± 0.19	5.86 ± 0.95
**2**	> 10	> 10
**LY294002** ^b^	1.06 ± 0.06	3.85 ± 1.25

^a^ Concentration necessary for 50% inhibition (IC_50_); results are presented as mean ± S.E.M. (*n* = 3). ^b^ LY294002 (2-morpholin-4-yl-8-phenylchromen-4-one) was used as a reference compound.

## Supplementary Materials

Supplementary materials Figure S1–S14 are available online.
