# Supplementary material for: New Marine Sterols from a Gorgonian Pinnigorgia sp
Source: Molecules. 2017 Mar 3;22(3):393. doi: 10.3390/molecules22030393 (PMC6155433; doi:10.3390/molecules22030393)
Supplement: Supplementary file 1 [file molecules-22-00393-s001.pdf]

Supporting Information

New Marine Sterols from a Gorgonian *Pinnigorgia* sp.

Yu-Chia Chang , Tsong-Long Hwang , Chih-Hua Chao , Ping-Jyun Sung *

No	Content	page
S1.	HRESIMS spectrum of compound **1**.	2
S2.	^{1}H NMR spectrum (400 MHz) of compound **1** in $CDCl_3$.	2
S3.	^{13}C NMR spectrum (100 MHz) of compound **1** in $CDCl_3$.	3
S4.	gHSQC spectrum (400 MHz) of compound **1** in $CDCl_3$.	3
S5.	^{1}H–^{1}H COSY spectrum (400 MHz) of compound **1** in $CDCl_3$.	4
S6.	gHMBC spectrum (400 MHz) of compound **1** in $CDCl_3$.	4
S7.	NOESY spectrum (400 MHz) of compound **1** in $CDCl_3$.	5
S8.	HRESIMS spectrum of compound **2**.	5
S9.	^{1}H NMR spectrum (400 MHz) of compound **2** in $CDCl_3$.	6
S10.	^{13}C NMR spectrum (100 MHz) of compound **2** in $CDCl_3$.	6
S11.	gHSQC spectrum (400 MHz) of compound **2** in $CDCl_3$.	7
S12.	^{1}H–^{1}H COSY spectrum (400 MHz) of compound **2** in $CDCl_3$.	7
S13.	gHMBC spectrum (400 MHz) of compound **2** in $CDCl_3$.	8
S14.	NOESY spectrum (400 MHz) of compound **2** in $CDCl_3$.	8

Mass Spectrum SmartFormula Report

Analysis Info

Analysis Name	D:\Data\a2\2012PINEAM030502081206_000002.d	12/9/2014 3:09:35 PM
Method	broadband first signal	
Sample Name	2012PinEA-M03-05-02-08-12-06	Instrument: FT-MS solariX
Comment	ESI Positive	

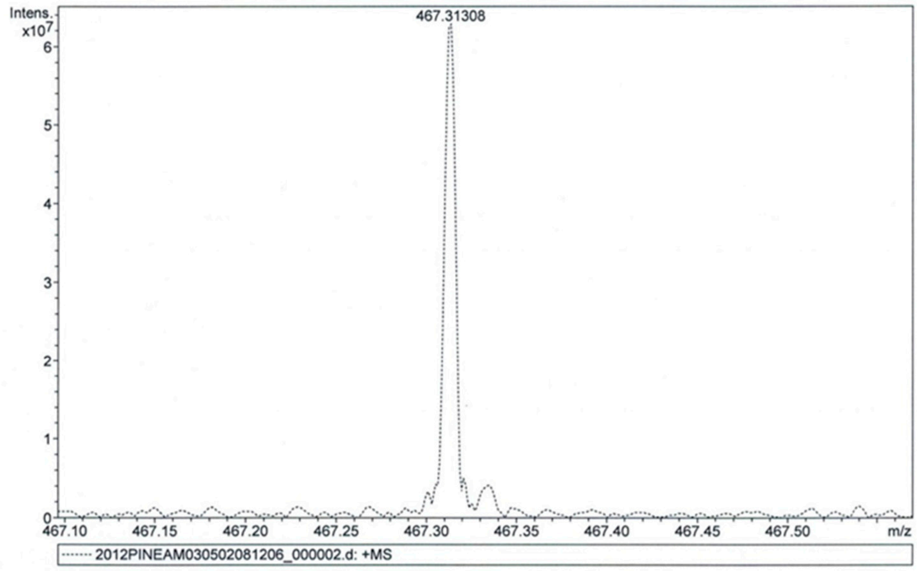

Meas. m/z	#	Formula	Score	m/z	err [mDa]	err [ppm]	mSigma	rdb	e⁻ Conf	N-Rule
467.31308	1	C 28 H 44 Na O 4	100.00	467.31318	0.11	0.23	31.0	6.5	even	ok

S1. HRESIMS spectrum of compound **1**.

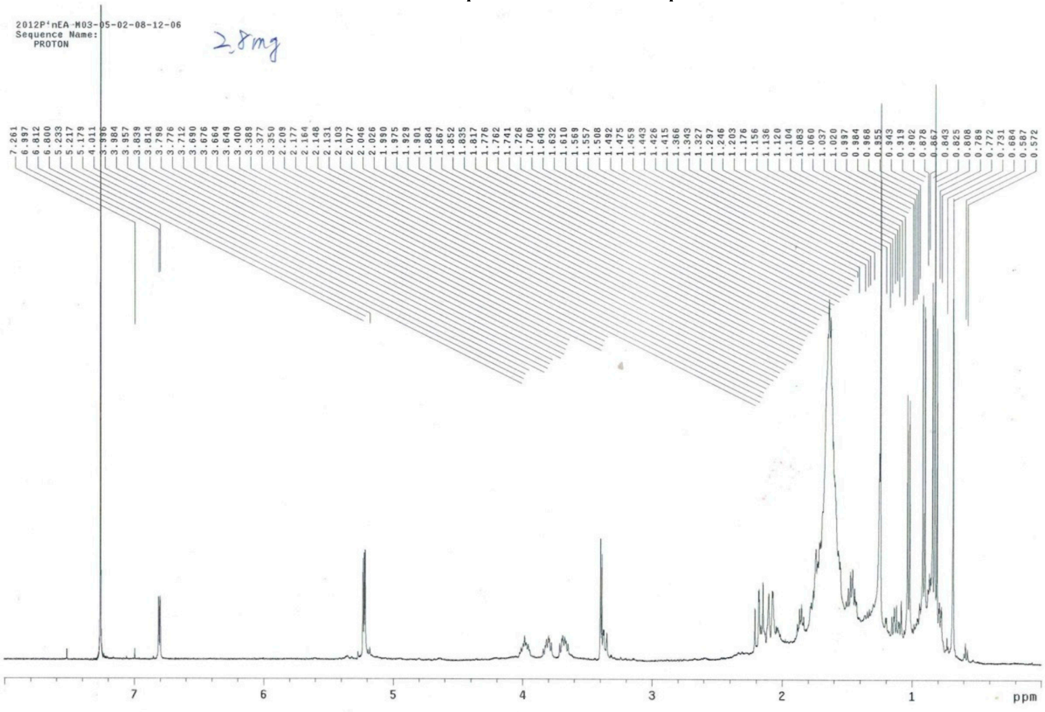

S2. ^{1}H NMR spectrum (400 MHz) of compound **1** in $CDCl_3$.

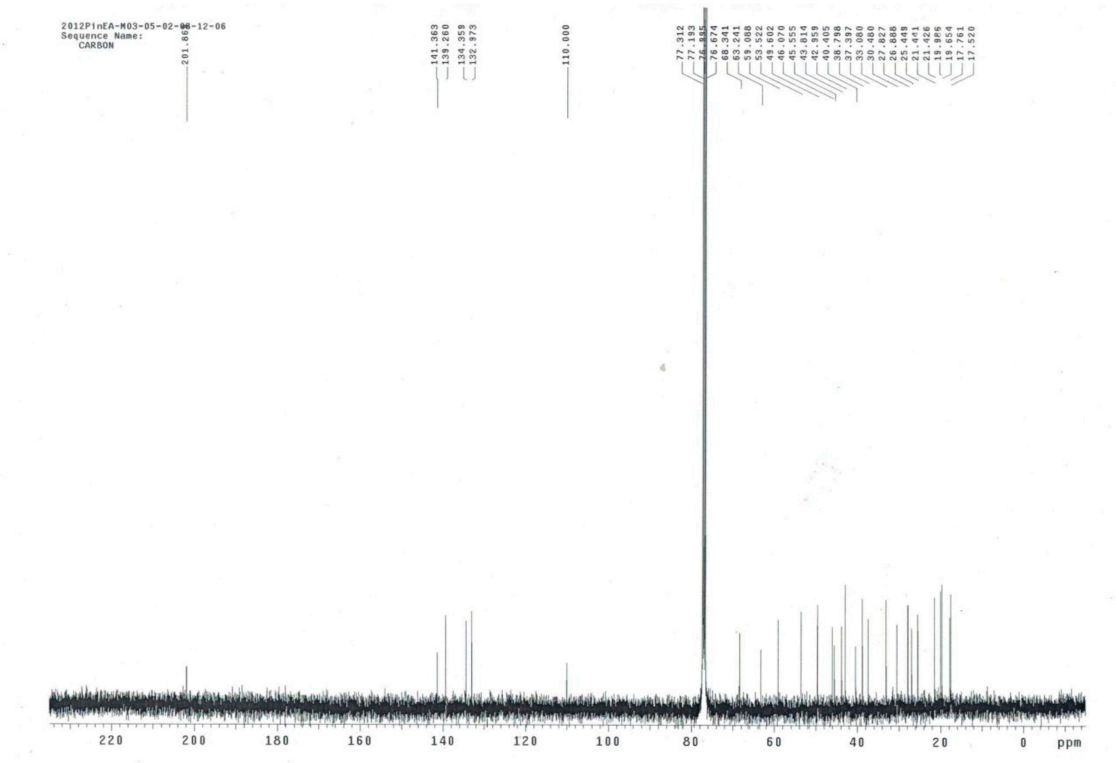

S3. ^{13}C NMR spectrum (100 MHz) of compound **1** in $CDCl_3$.

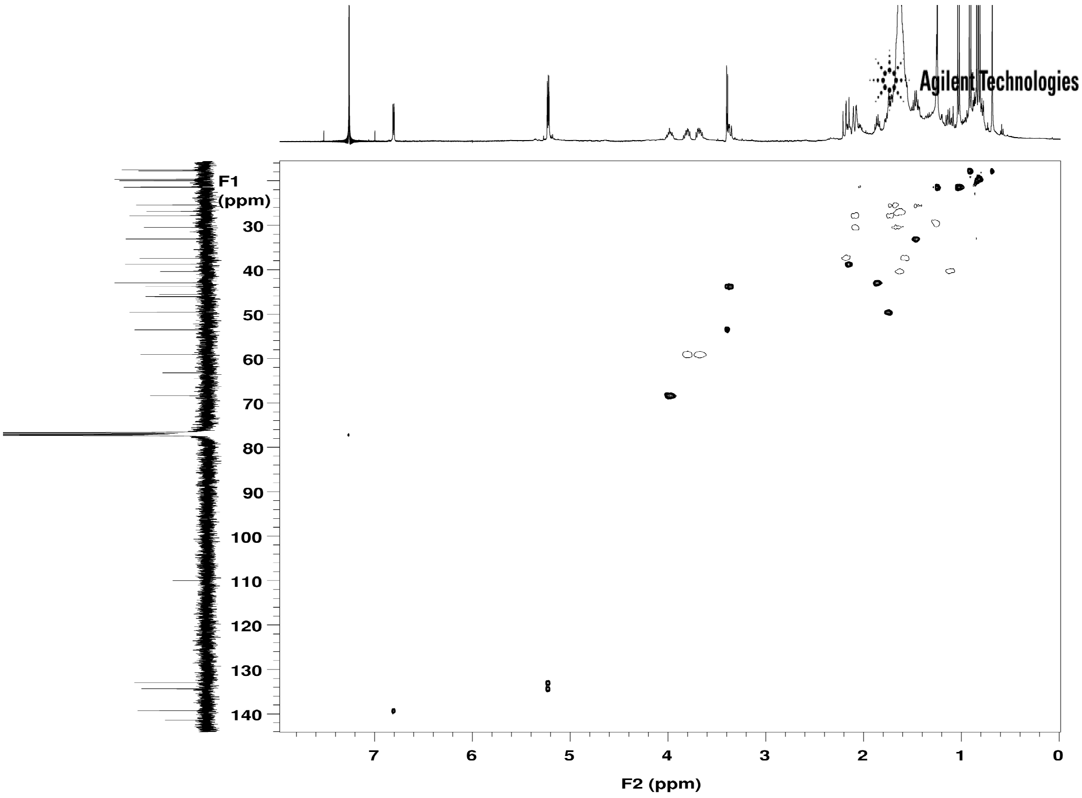

S4. gHSQC spectrum (400 MHz) of compound **1** in $CDCl_3$.

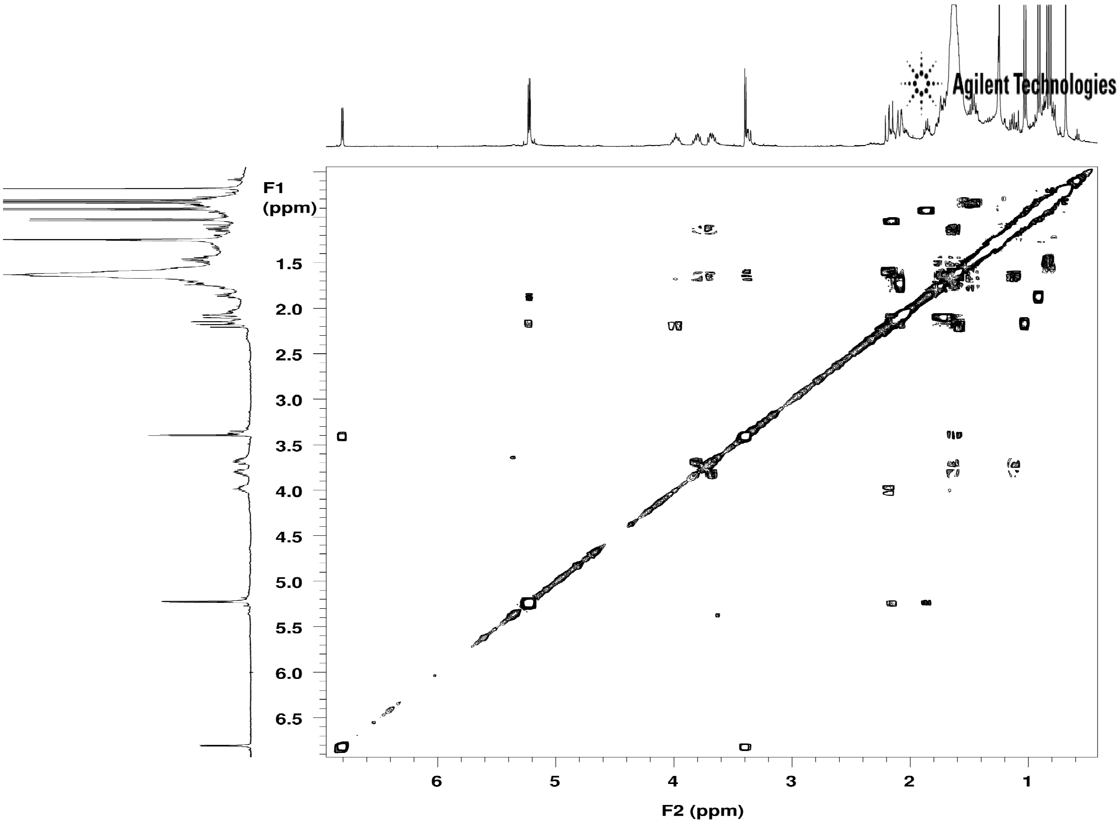

S5. ^{1}H–^{1}H COSY spectrum (400 MHz) of compound **1** in $CDCl_3$.

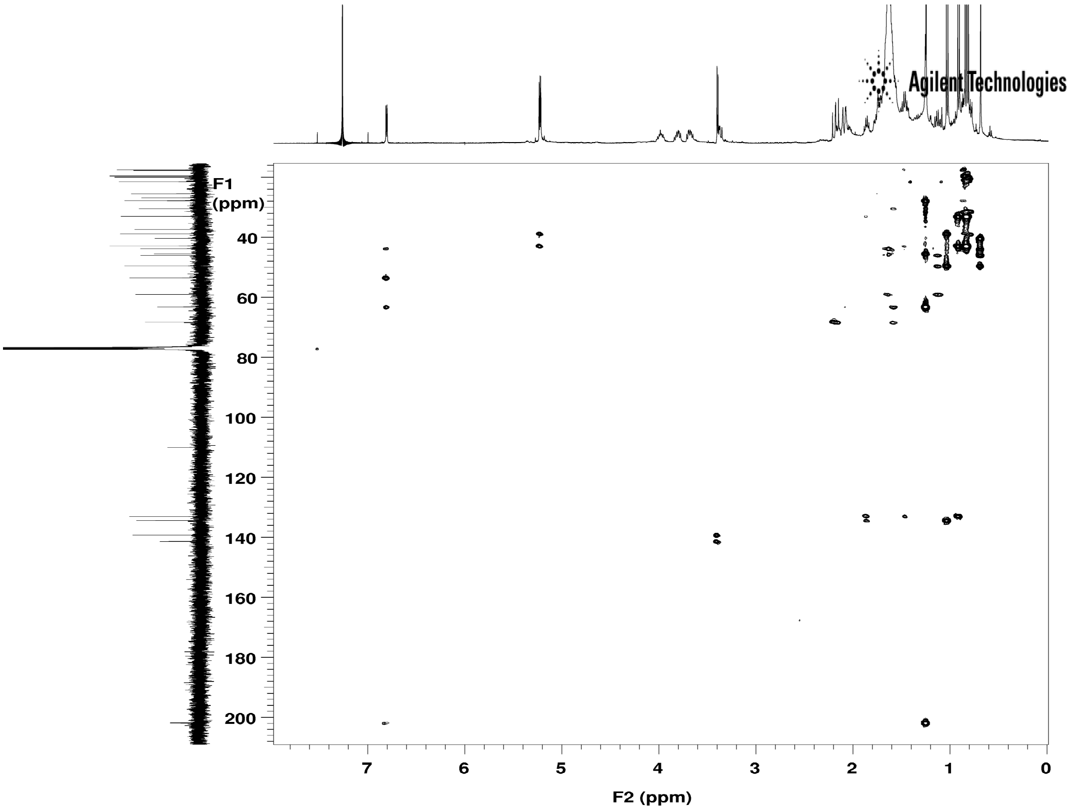

S6. gHMBC spectrum (400 MHz) of compound **1** in $CDCl_3$.

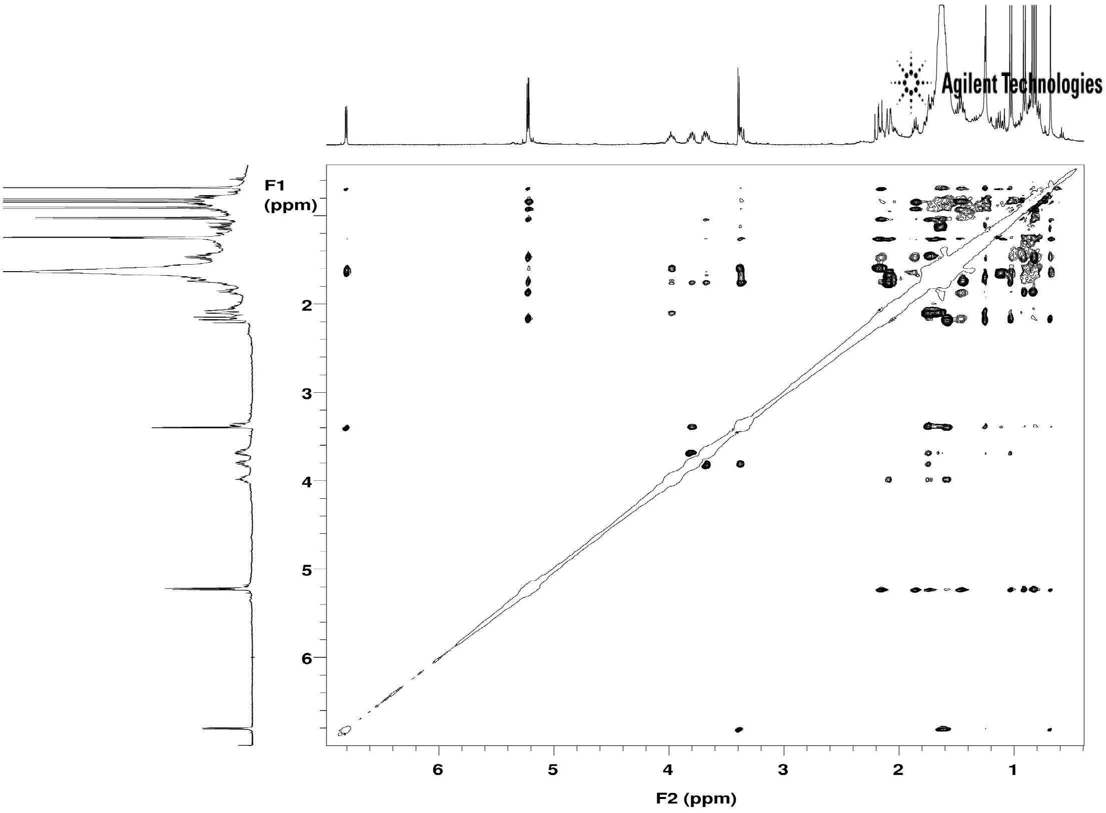

S7. NOESY spectrum (400 MHz) of compound **1** in $CDCl_3$.

Mass Spectrum SmartFormula Report

Analysis Info

Analysis Name	D:\Data\a2\2012pineam030502041204_000002.d	6/5/2014 3:22:02 PM
Method	broadband first signal	
Sample Name	2012PinEA-M03-05-02-04-12-04	Instrument: FT-MS solariX
Comment	ESI Positive	

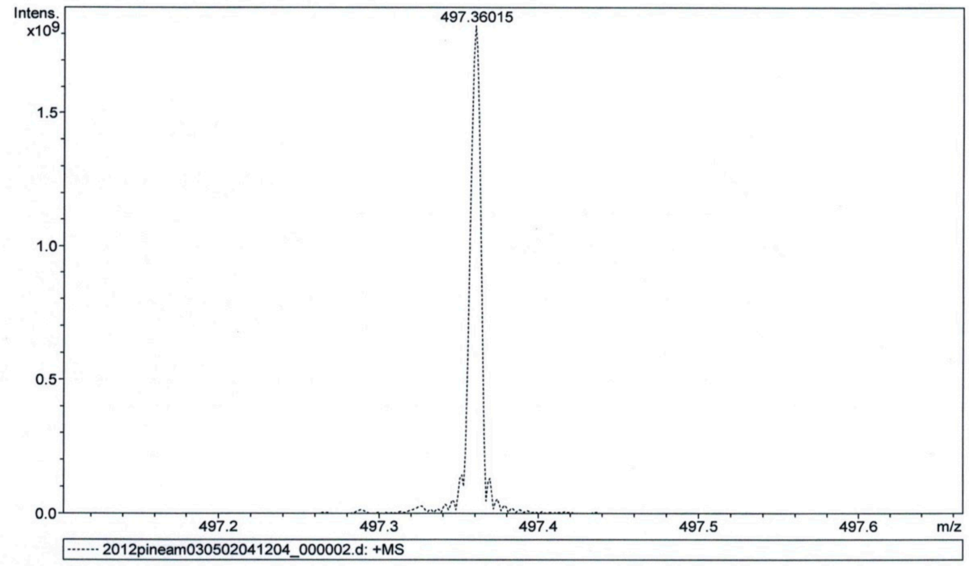

Meas. m/z	#	Formula	Score	m/z	err [mDa]	err [ppm]	mSigma	rdb	e⁻ Conf	N-Rule
497.36015	1	C 30 H 50 Na O 4	100.00	497.36013	-0.02	-0.03	13.5	5.5	even	ok

S8. HRESIMS spectrum of compound **2**.

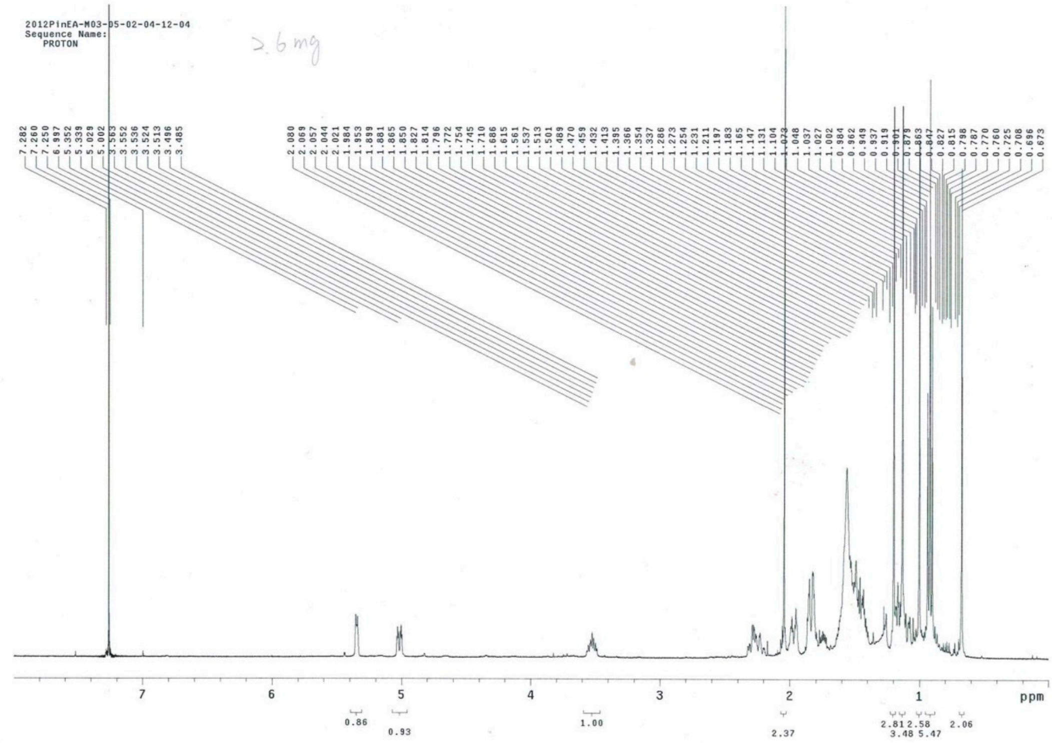

S9. ^{1}H NMR spectrum (400 MHz) of compound **2** in $CDCl_3$.

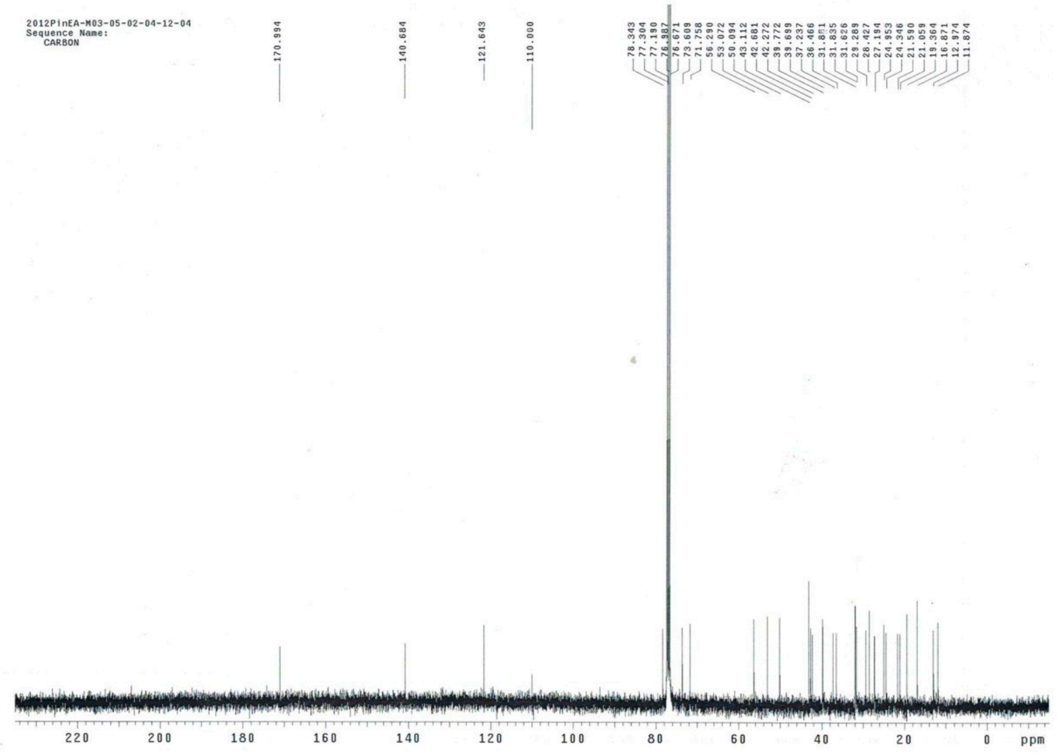

S10. ^{13}C NMR spectrum (100 MHz) of compound **2** in $CDCl_3$.

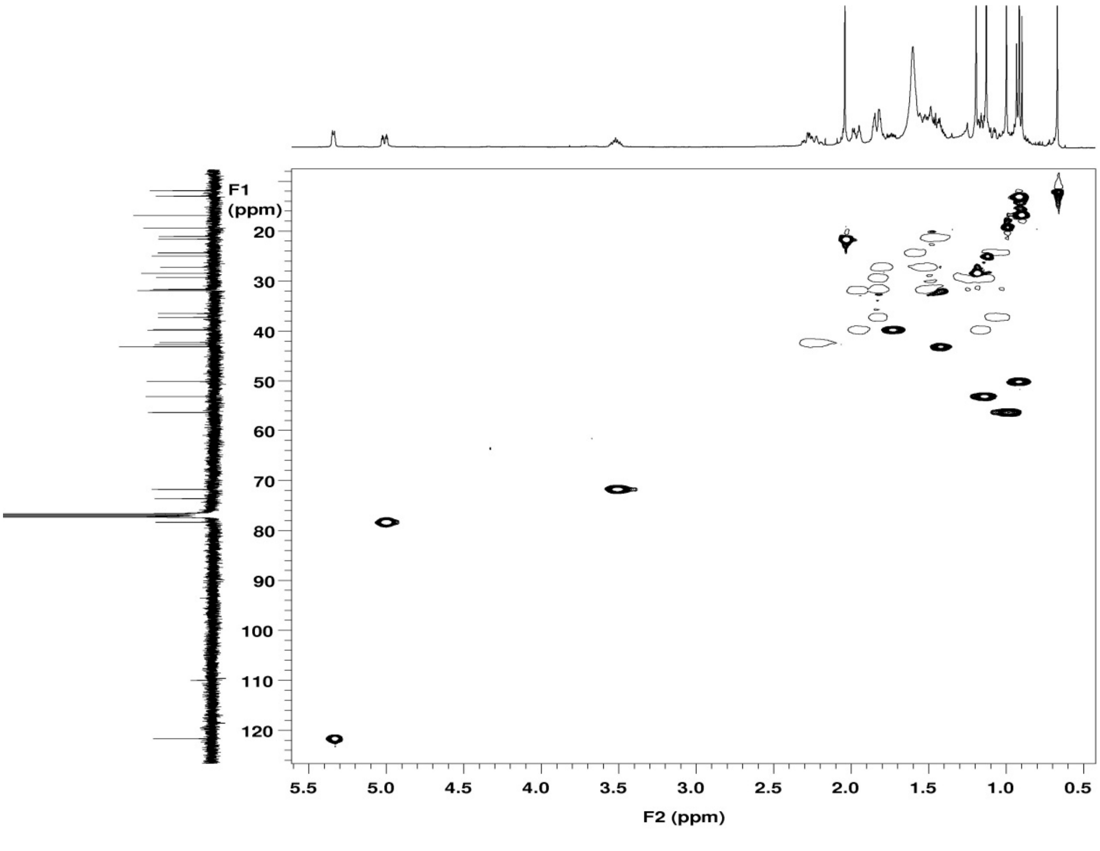

S11. gHSQC spectrum (400 MHz) of compound **2** in $CDCl_3$.

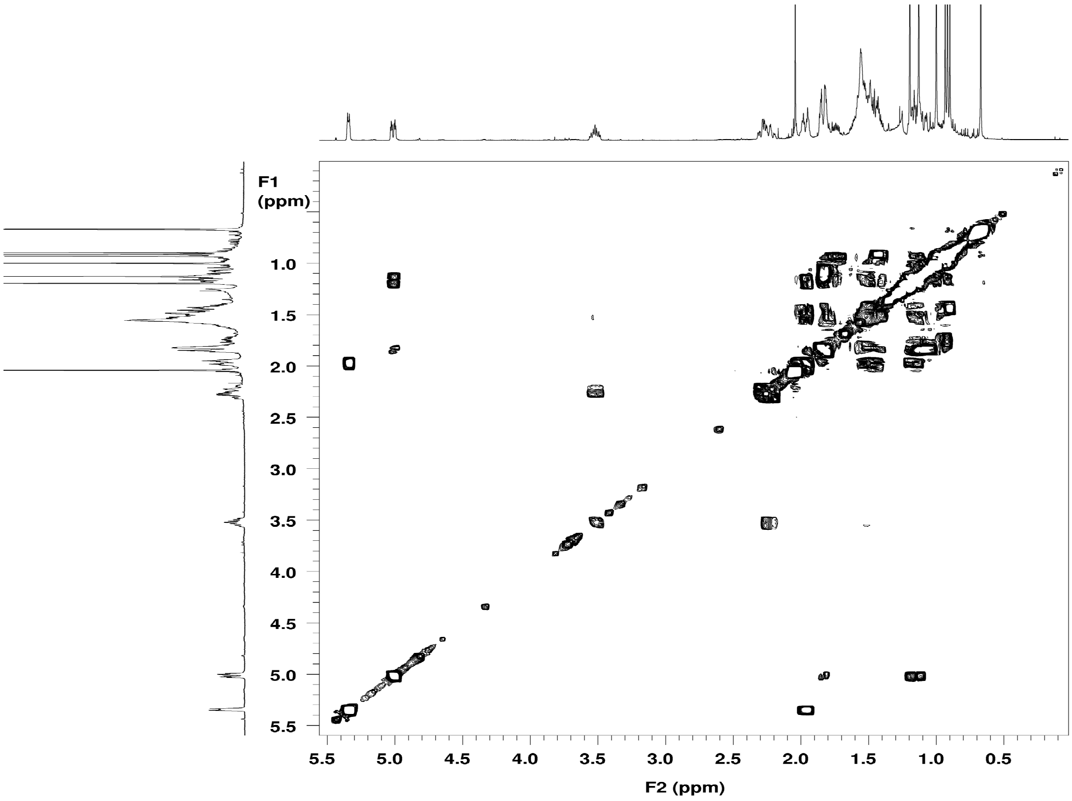

S12. $^{1}H-^{1}H$ COSY spectrum (400 MHz) of compound **2** in $CDCl_3$.

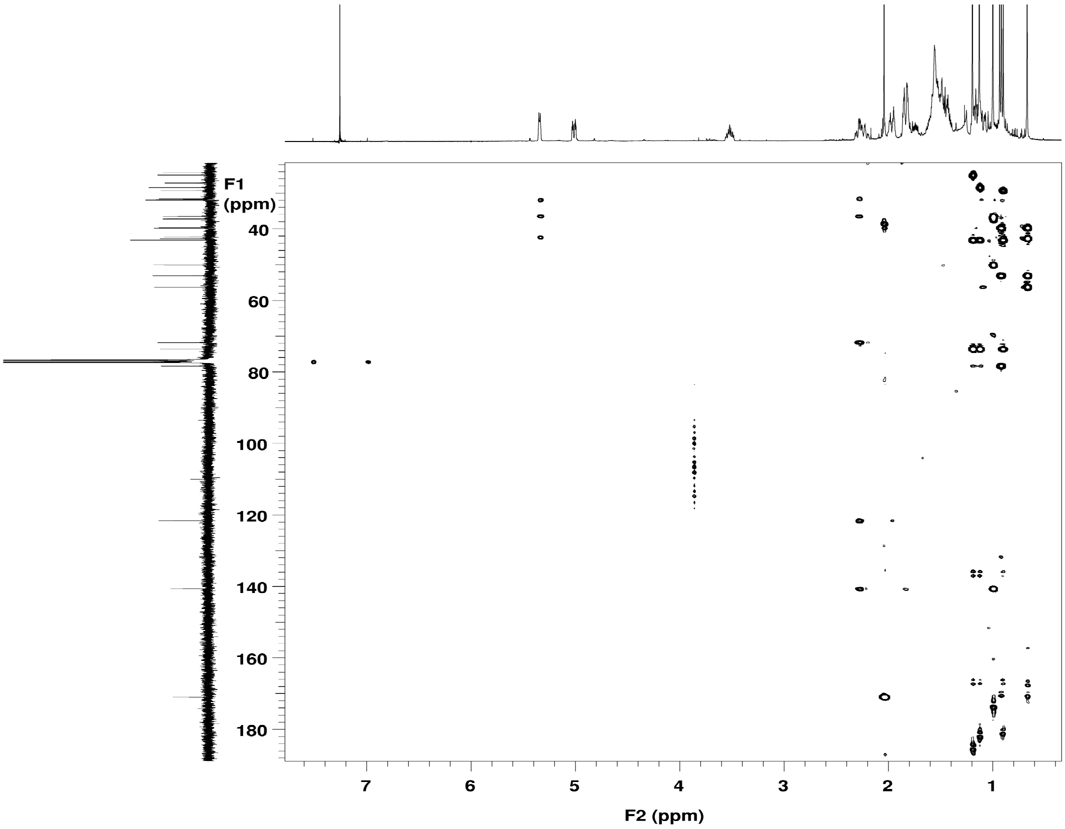

S13. gHMBC spectrum (400 MHz) of compound **2** in $CDCl_3$.

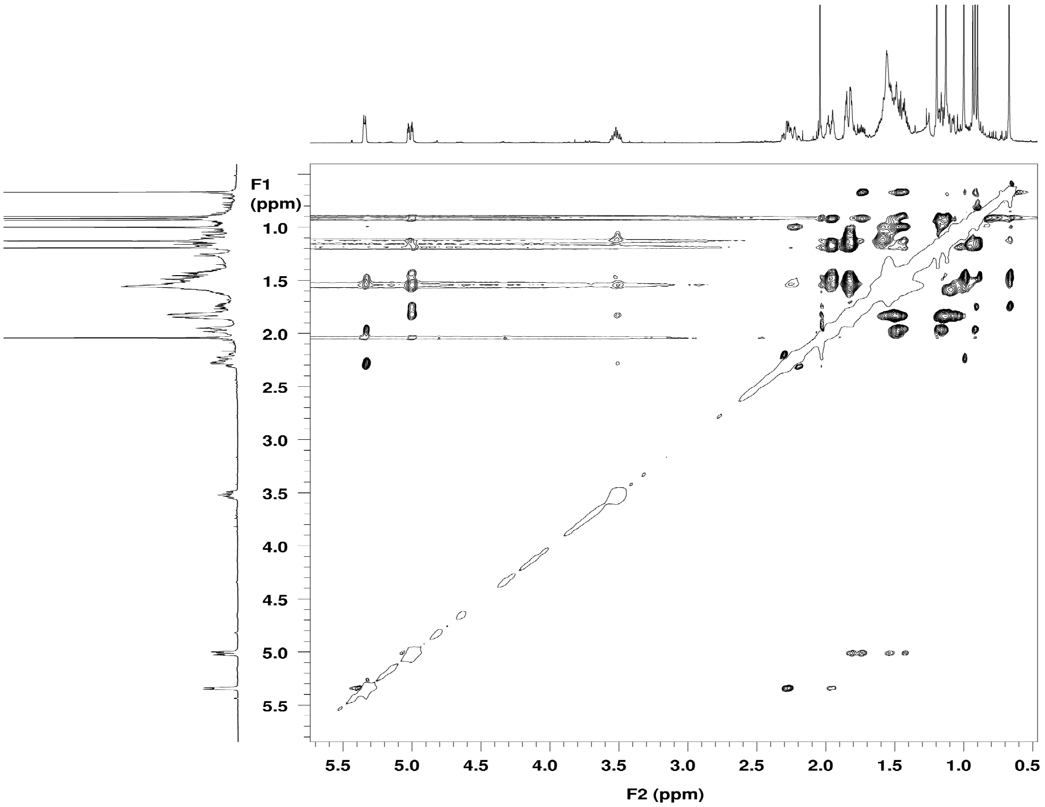

S14. NOESY spectrum (400 MHz) of compound **2** in $CDCl_3$.
